# Aqueous Extract of *Lysimachia christinae* Hance Prevents Cholesterol Gallstone in Mice by Affecting the Intestinal Microflora

**DOI:** 10.4014/jmb.2106.06043

**Published:** 2021-07-15

**Authors:** Shijia Liu, Quji Luorong, Kaizhi Hu, Weiguo Cao, Wei Tao, Handeng Liu, Dan Zhang

**Affiliations:** 1 First Clinical College, Chongqing Medical University, Chongqing 400016, P.R.China; 2 College of Traditional Chinese Medicine, Chongqing Medical University, Chongqing 400016, P.R.China; 3 Chongqing Institute of Pharmaceutical Plant, Chongqing 408435, P.R.China; 4 Laboratory of Tissue and Cell Biology, Experimental Teaching Center, Chongqing Medical University, Chongqing 400016, P.R.China; 5 Molecular Medicine and Cancer Research Center, Department of Cell Biology and Genetics, Chongqing Medical University, Chongqing 400016, P.R.China

**Keywords:** *Lysimachia christinae* Hance, traditional Chinese medicine, cholesterol cholelithiasis, intestinal microflora

## Abstract

With changes in human dietary patterns, the proportion of high-fat and high-cholesterol foods in the daily diet has increased. As a result, the incidence rate of cholelithiasis is increasing rapidly. Many studies have reported on the crucial role that the intestinal microflora plays in the progression of gallstones. Although the whole herb of *Lysimachia christinae*, a traditional Chinese medicine, has long been extensively used as a remedy for cholelithiasis in China, its effects on the intestinal microflora remain unknown. Hence, in this study, we investigated the ability of the aqueous extract of *L. christinae* (LAE) to prevent cholesterol gallstones (CGSs) in model animals by affecting the intestinal microflora. The effects of LAE on body weight, serum lipid profile, visceral organ indexes, and histomorphology were studied in male C57BL/6J mice, which were induced by a lithogenic diet. After the 8-week study, CGSs formation was greatly reduced after LAE treatment. LAE also reduced body weight gain and hyperlipidemia and restored the histomorphological changes. Moreover, the intestinal microflora exhibited significant variation. In the model group fed the lithogenic diet, the abundances of the genera unclassified *Porphyromonadaceae*, *Lactobacillus* and *Alloprevotella* decreased, but in contrast, *Akkermansia* dramatically increased compared with the control check group, which was fed a normal diet; the administration of LAE reversed these changes. These results imply that *L. christinae* can be considered an efficient therapy for eliminating CGSs induced by a high-fat and high-cholesterol diet, which may be achieved by influencing the intestinal microflora.

## Introduction

Gallstone disease has long been one of the most common afflictions of the digestive system. Especially in recent years, the incidence rate of gallstones has been increasing with changes in peoplés lifestyle and diet. Patients with gallstones are among the most frequently admitted patients in European hospitals. The socioeconomic costs of gallstone disease have also been rising. In developed countries, approximately 20% of adults have gallstones, and morbidity has been increasing by 0.60%-1.39% per year [1]. The incidence of cholelithiasis in adults is approximately 10% of the total population and as high as 15% among middle-aged women [2]. In Western countries, approximately 80% of gallstones are cholesterol gallstones (CGSs), which are mainly formed by cholesterol and are caused by an exceedingly high level of cholesterol in bile or serum [3]. Generally, the clinical treatment of gallstone disease includes cholecystectomy, lithotripsy, endoscopy, or the administration of drugs such as nonsteroidal anti-inflammatory drugs (NSAIDs) or ursodeoxycholic acid (UDCA). Unfortunately, the therapies mentioned above have some limitations. Cholecystectomy carries a small but existing complication rate, and the efficiency of medical therapy is far from satisfactory. Therefore, there is an increasing demand for a preferable method to prevent CGSs.

*Lysimachia christinae* Hance (*L. christinae*) is a leguminous plant that is distributed widely in temperate climates, especially in China[4]. In traditional Chinese medicine, this herb has the effects of clearing away heat, removing dampness and diuresis. It is commonly used as a therapy for diseases such as jaundice and lithiasis in clinical practice.

The causes of cholecystolithiasis are complicated. Variation in the intestinal microflora has been an important research hotspot in recent years [5-7]. Although *L. christinae* has been frequently used for cholelithiasis in the clinic, there is no experimental report relevant to its ability to prevent gallstones by affecting the intestinal microflora until now. Previous studies [2, 8-10] have paid more attention to the changes in bile composition but did not involve the intestinal microflora. Therefore, it is necessary to investigate the changes in intestinal microflora after the administration of *L. christinae*. In this paper, the effects of *L. christinae* on preventing gallstones in mice fed a lithogenic diet were evaluated, as were the changes in the intestinal microflora.

## Materials and Methods

### Chemicals

Feed constituting a lithogenic diet (containing 10% fat, 1.25% cholesterol and 0.5% bile salt) was purchased from Nantong Trophic Animal Feed High-tech Co., Ltd. A total bile acid (TBA) assay kit (E003-2-1), a total cholesterol (TC) assay kit (A111-1-1), a triglyceride (TG) assay kit (A110-1-1), an aspartate aminotransferase (AST) assay kit (C010-2-1), an alanine aminotransferase (ALT) assay kit (C009-2-1), an alkaline phosphatase (AKP) assay kit (A059-2), a low-density lipoprotein cholesterol (LDL-C) assay kit (A113-1-1), and a high-density lipoprotein cholesterol (HDL-C) assay kit (A112-1-1) were purchased from Nanjing JianCheng Bioengineering Institute. An E.Z.N.A.™ Mag-Bind Soil DNA Kit (M5635-02) was purchased from Omega Bio-Tek. A Qubit3.0 DNA detection kit (Q10212) was bought from Life. Hieff^®^ Robust PCR Master Mix (2×; 10105ES03) and Hieff NGS™ DNA Selection Beads (12601ES56) were purchased from Yeasen.

### *L. christinae* Aqueous Extract (LAE)

*L. christinae* samples were collected from Anyue, Sichuan, China, in September 2020. The samples were dried in an electric drying oven at 70°C with forced convection. Given that *L. christinae* is taken orally in the form of a water decoction in traditional Chinese medicine, an aqueous extract of *L. christinae* was prepared. Dried *L. christinae* Hance was extracted with tap water three times. The first time, it was boiled for 2 h with 15 times of water. Subsequently, the sample was boiled for 2 h with 12 times of water and then for 2 h with 10 times of water. The extraction solution was first filtered by using gauze and then vacuum suction filtration. The entire filtered solution of *L. christinae* was concentrated under normal atmospheric pressure at 55°C. Finally, the concentrate was lyophilized, and the product was stored at -20°C.

### Animals

Six-week-old male C57BL/6J mice weighing 18–22 g were purchased from Chongqing Ensiweier Biotechnology Co., Ltd. and raised under controlled conditions (24 ± 0.5°C and 12 h of light from 7:00 a.m. to 7:00 p.m.) in the Central Animal House of Chongqing Medical University. All animals were housed in polypropylene cages (5 mice per cage) with sawdust as bedding and provided ad libitum access to food and water.

The animal experiments were approved by the Animal Research Welfare Committee of Chongqing Medical University, China, and were periodically inspected by the Chongqing Laboratory Animal Care and Use Committee. The experiments were carried out following the National Research Council Guide for the Care and Use of Laboratory Animals (National Research Council, 1996).

### Animal Treatment

Sixty mice were randomly divided into 5 groups by using a random number table. After acclimating for one week, mice from the above 5 groups were treated as follows: the control check group (CK) were supplied a normal diet and saline; the model group (M) were fed a lithogenic diet and saline as negative control; and while administering the lithogenic diet, 3 *L. christinae* treatment groups (JL, JM, JH) were given suspensions of LAE in pure water at a low dose (370 mg/ml), middle dose (556 mg/ml) or high dose (830 mg/ml), respectively. The mice received their respective treatment by gavage at the dosage of 0.1 ml/10 g body weight once a day. The body weights of all mice before the start of the experiment were recorded. The duration of LAE intervention was 8 weeks. At the end of treatment, fecal samples were collected from the mice with disposable capped sterile centrifuge tubes. After sampling, the feces were quickly put into liquid nitrogen for quick freezing and then transferred to a -80°C freezer for storage; the samples were stored in dry ice throughout transportation. All mice were fasted for 12 h before the end of the experiment but given free access to water. One hour after the final administration of treatment, all animals were weighed and anesthetized. Then, blood was taken through the orbital venous plexus of each mouse and centrifuged (1,700 × *g*, 15 min, 4°C) to separate serum for biochemical analysis. Each abdominal cavity was opened along the medial line. The gallbladders were cut, weighed and observed for the presence of gallstones. Bile was collected from each gallbladder by syringe and stored in Eppendorf tubes at -20°C for further analysis. Thereafter, the livers were quickly excised, trimmed of afﬁliate tissue, washed with ice-cold PBS, blotted dry, weighed, and ﬁxed in 4% neutral buffered formaldehyde for subsequent experiments.

### Biochemical Analyses of the Bile and Blood

Serum and bile were diluted appropriately before measuring the biochemical indexes. A test kit was used to determine the contents of ALT, AST, ALP, TC, HDL-C, LDL-C, TG, and TBA in the serum samples. The TBA and TC levels in the bile were assayed with the same methods as used to determine those in serum.

### Histological Examination of the Liver

The liver samples ﬁxed with formaldehyde were embedded in parafﬁn, cut into 5-μm thick slices, stained with hematoxylin-eosin and examined by light microscopy.

### 16S rRNA Sequence Analysis

The feces of the mice were snap-frozen in liquid nitrogen before preservation at -80°C. The DNA of the total bacteria in the feces was extracted by using the E.Z.N.A. Mag-Bind Soil DNA Kit (Omega Bio-Tek, USA) according to the manufacturer’s instructions, qualified by 2% agarose gel electrophoresis, and quantified by a Qubit 3.0 fluorophotometer (Q32866, Invitrogen, USA). The V3–V4 hypervariable regions of the bacterial 16S rRNA gene were amplified with primers 341 F (5′-CCTACGGGNGGCWGCAG-3′) and 805 R (5′-GACTACHVGGGTATC-TAATCC-3′) by a polymerase chain reaction (PCR) system (ETC 811, Eastwin, China). The PCR products were purified by Hieff NGS DNA Selection Beads, quantified by using a Qubit 3.0 fluorophotometer, pooled in equivalent amounts, and sequenced using an Illumina MiSeq instrument (Illumina, USA). The Usearch 11.0.667 software package was used to assemble, control quality and cluster the obtained reads[11]. Taxonomy assignment was performed with the help of Silva database v128 [12] and the Naïve Bayesian Classifier from the Ribosomal Database Project (RDP) [13] with a bootstrap confidence cutoff of 70%.

### Statistical Analysis

Statistical analysis was done using the SPSS software program version 26.0. The data were expressed as mean ± SD and were analyzed with one-way ANOVA followed by the Bonferroni test or Dunnett T3 test. Differences were considered statistically significant for probability (*p* < 0.05).

Alpha diversity metrics were calculated using Mothur 1.43.0 software. Beta diversity analysis was performed by PCA using R 3.6.0 (The R Foundation Conference Committee). Microbial microflora composition analysis was performed using R 3.6.0. Cluster differences were tested by permutational multivariate analysis of variance (PERMANOVA) using “adonis” in package “vegan” in R 3.6.0.

## Results

### The Formation of Gallstones in Different Groups of Mice

As shown in Table 1, in the control group, no yellow granular gallstones were observed in the gallbladders of any of the 12 mice, and the gallstone formation rate was 0%. In the model group fed the lithogenic diet, gallstones were successfully induced and could be seen through the gallbladder walls of all 12 mice; the formation rate was 100%. The gallstones presented as white spherical or ellipsoidal aggregates (white/light yellow) and floated in the bile. In the remaining 3 groups of mice treated with LAE, gallstones were still observed in the gallbladders of each mouse. Although cholelithiasis was not eliminated by the administration of LAE, with increasing *L. christinae* concentration, the number of gallstones gradually decreased. These results show that *L. christinae* can reduce the extent of gallstones and that the cholagogic effect of LAE was dose-dependent.

### Body Weight Gain and Visceral Indexes

Although the initial body weights did not differ among the groups, the model mice gained more weight than the control check mice at the end of the experiment. LAE remarkably reduced body weight gain induced by the lithogenic diet, and this effect showed dose dependence (*p* < 0.05 and 0.01) (Table 2).

A similar phenomenon was observed for the weight changes of the internal organs. The livers and gallbladders of the model group were heavier than those of the control check group. The administration of LAE reversed this change, especially at the high dose (*p* < 0.05 and 0.01). The above results were then confirmed by calculating the visceral index (Table 2).

### Histomorphology of the Liver

**Fig. 1:**
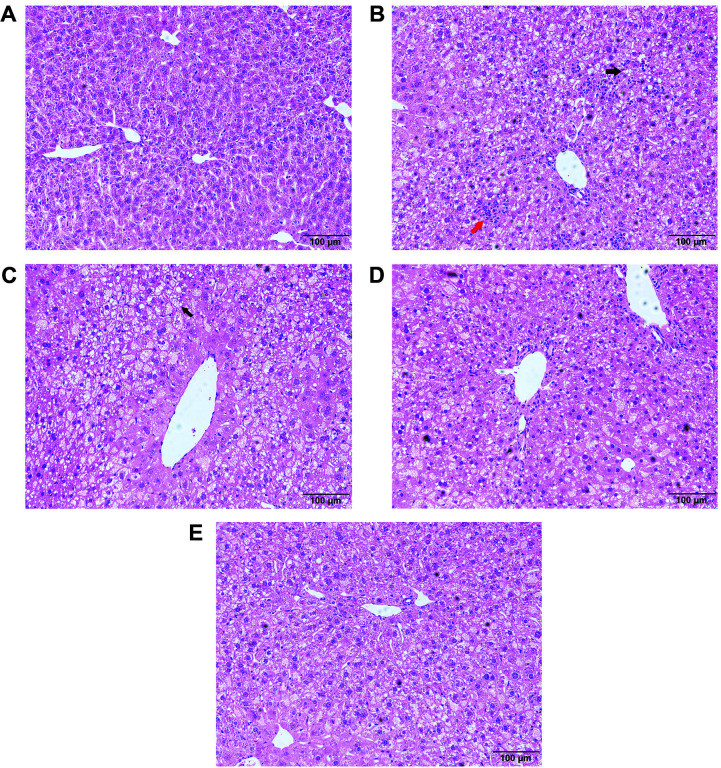
Histological structure of liver in each group. (**A**) control check; (**B**) model group (treated with saline); (**C**) low dose of LCAE group; (**D**) middle dose of LCAE group; (**E**) high dose of LCAE group. Black arrowheads indicate lipid droplets in hepatocytes, and red arrowheads indicate fibrous hyperplasia.

Histological examination showed fatty degeneration of the liver in mice fed a high-fat and high-cholesterol diet, which manifested as ballooning degeneration of the liver cells, accumulation of lipid droplets and the appearance of fibrous hyperplasia (Fig. 1B). LAE showed a dose-dependent recovery effect and could restore these pathological changes to a certain extent, especially at the high dose. In mice treated with LAE, the liver cells had less lipid accumulation and fibrous hyperplasia (Figs. 1C-1E).

### Lipid Levels of the Plasma

Compared with the control check group, the TC, TG, LDL-C and TBA plasma levels in the model group induced by lithogenic diet were significantly increased while the level of HDL-C decreased, which indicated that the CGS model was successfully established. Administration of LAE restored the concentrations of TC, TG, LDL-C and HDL-C in plasma and enhanced the secretion of TBAs. Although the effects of low-dose LAE were not satisfactory, high-dose LAE could restore the plasma TG, LDL-C and HDL-C levels of model mice to almost the normal levels (Table 3).

### Lipid Levels of the Bile

As shown in Table 4, a lithogenic diet led to a large increase in TC and TBA contents in bile. LAE displayed a dose-dependent decrease in the TC content and remarkably promoted the secretion of TBA into bile.

### Biochemical Markers of Liver Injury

The data in Table 5 indicated that the plasma levels of ALT, AST, and AKP in the model group were elevated by the lithogenic diet. The usage of LAE could reverse this trend to varying degrees. Plasma levels of the above three markers in all LAE treatment groups were lower than those in the model group, although the differences were not statistically significant.

### Changes in Intestinal Microflora

**Fig. 2:**
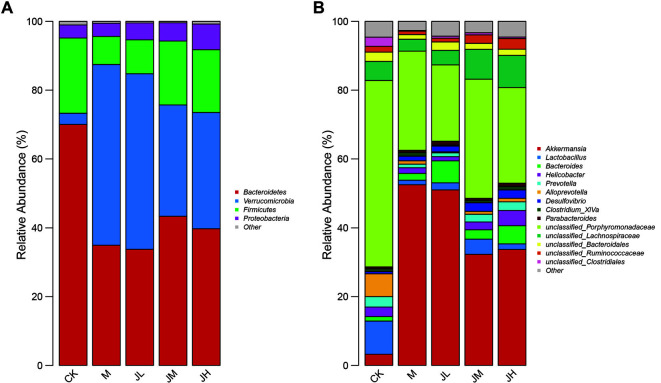
Bar charts of the relative abundance of the bacterial community of phylum (**A**) and genus (**B**) levels in different groups of mice.

The relative abundances of the intestinal microflora per group were shown in Fig. 2, and to show the differences in composition more clearly, the detailed numbers of the abundances were shown in Table 6 and 7. At the phylum level, *Bacteroidetes* was the primary intestinal microflora in the CK group but its contents decreased in the model group. The lithogenic di*et al*so led to an increase in *Verrucomicrobia* and a decrease in *Firmicutes*. The usage of LAE reversed this phenomenon to a certain extent (Fig. 2A and Table 6). At the genus level, the dominant component in the CK group was *unclassified*_*Porphyromonadaceae*, while its content decreased in the model group. Moreover, the proportions of *Lactobacillus* and *Alloprevotella* were also reduced by the lithogenic diet. In contrast, the number of *Akkermansia* increased in the model group. LAE exhibited similar dose-dependent reversal effects (Fig. 2B and Table 7). The proportion of the main intestinal microflora mentioned above in the JL group was close to that in the M group. In contrast, the relative abundances of the main intestinal microflora in the JM and JH groups were close to that in the CK group, especially the phylum *Firmicutes*.

**Fig. 3:**
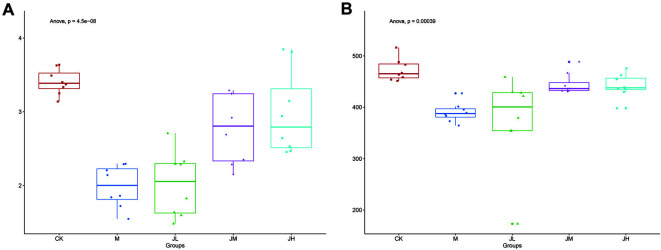
Alpha diversity in fecal samples of mice. Box plots showing alpha diversity in samples using the species richness estimators Shannon (**A**) and ACE (**B**) index.

The Shannon index and ACE index were adopted to evaluate the alpha diversity (Fig. 3). The Shannon index (Fig. 3A) and ACE index (Fig. 3B) decreased remarkably in the model group. The application of LAE could restore the alpha diversity of intestinal microflora.

**Fig. 4:**
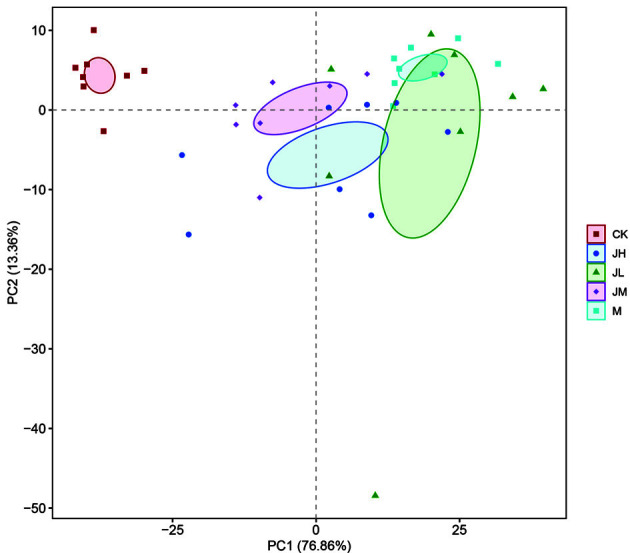
Alpha diversity in fecal samples of mice.

Fig. 4 showed the beta diversity results of the different groups. We used the PCA analysis to investigate the similarity of intestinal microflora between different samples. The results demonstrated that the model group was separate from the CK group, and after LAE gavage, this change was partially reversed. The compositions of the intestinal microflora in the JM and JH groups were closer to that in the CK group than to that in the model group. Furthermore, Adonis analysis (Table 8) confirmed that the composition differences between the JM group and the model group and between the JH group and the model group were considered statistically significant (*p* < 0.05). In addition, all of these effects of LAE were dose-dependent. The high and middle doses of LAE exhibited more noteworthy therapeutic effects than the low dose.

## Discussion

In recent years, gallstones with cholesterol as the principal component have become the main type of cholelithiasis in China. The formation of cholesterol gallstones is a complex and multifactorial process, and its pathogenesis is still not fully understood. Many studies on this issue have been conducted and the change in bile components secreted by the liver was shown to be the primary reason for the formation of gallstones [14]. When the intake of high-cholesterol foods is excessively high and goes beyond the regulatory capacity of the liver, the absolute overdose amount of cholesterol in the bile will result in cholesterol supersaturation and become thermodynamically unstable "lithogenic bile," causing cholesterol to precipitate in a crystalline form and form cholesterol stones.

At present, many studies have shown that there is an important relationship between the intestinal microflora and metabolic diseases of the human body [15-19]. Many diseases, such as Crohn's disease [20], colon cancer [21], and metabolic diseases [22, 23], have been proven to be related to the intestinal microflora. A large number of bacteria colonize the normal human intestinal tract and are collectively called the intestinal microflora. It is composed of 30 genera and 500 species. The total number of bacteria can reach 10^14^, which is 10 times greater than the total number of human cells [24]. Intestinal microflora can not only promote food digestion but also protect the host from the invasion of pathogens and regulate metabolism [25]. Under normal circumstances, the intestinal microflora maintains a certain balance of quantity and proportion. When affected by changes in the host or external environment, this balance is broken, which will lead to disease. Therefore, it is of great significance to explore the mechanism of CGSs formation from the perspective of the intestinal microflora.

In this study, we adopted 16S rRNA sequencing to observe the composition change in the intestinal microflora after LAE treatment. The results showed that the intestinal microflora of cholelithiasis mice in the model group had notable changes in the species present and the quantity compared with the CK group. At the phylum level, the abundance of *Bacteroidetes* and *Firmicutes* decreased, while *Verrucomicrobia* increased in the model group. At the genus level, unclassified_*Porphyromonadaceae*, *Lactobacillus* and *Alloprevotella* decreased in the model group, but the number of *Akkermansia* increased dramatically. *Bacteroidetes* has already been revealed to be associated with lower cholesterol activity [26]. *Lactobacillus* is representative probiotic species [27], and its decrease makes the stability and physiological functions of the intestinal tract potentially dangerous and vulnerable to external deleterious factors. Previous studies have also found that the metabolites of *Lactobacillus* contribute to lowering cholesterol and activating its precipitation [28, 29]. *Verrucomicrobia* is a newly identified bacterial phylum, including a few recognized species, one of which is *Akkermansia*. The role of *Verrucomicrobia* in the mechanism of gallstone formation needs further study. Many previous studies have shown similar results [30-32], but here, there is something different: former studies have found that the most abundant phylum in all groups was *Firmicutes* [33]. In this study, *Bacteroidetes* was the primary intestinal microflora in the CK group, but *Verrucomicrobia* increased to reach almost the same level as *Bacteroidetes*, and these two phyla became dominant in the remaining four groups. From the alpha diversity analysis, the levels of the richness indicators (Shannon index and ACE index) showed that a lithogenic diet induced decreases in species richness. Both the Shannon and ACE indexes were lower in the M group than in the CK group, and after the administration of LAE, these indexes increased, especially in the JM and JH groups. The alpha diversity change results showed that LAE can improve the richness of the intestinal microflora. The PCA analysis showed that the compositions of intestinal microflora in the JM and JH groups were more similar to that in the CK group than to that in the model group, and the following Adonis analysis confirmed that this change was statistically significant. The above results suggest that the changes in intestinal microflora composition and diversity may be of great significance during the formation of gallstones. LAE treatment had a dose-dependent effect on the reversal of the intestinal microflora change caused by the lithogenic diet, which may be the mechanism by which LAE prevents gallstones.

The changes in other indicators were synchronous with the changes in the intestinal microflora. Through this research we also found that LAE could lower body weight gain and increase the visceral indexes induced by the lithogenic diet. Regarding the lipid profiles in the bile and serum, LAE induced a great decrease in serum TC, TG and LDL-C levels, as well as the TC content in the bile. In addition, LAE increased the level of HDL-C in serum and enhanced the secretion of TBAs. Additionally, LAE could partly recover the lipid degeneration in the liver induced by a high-fat and high-cholesterol diet. The above effects were all dose-dependent.

In conclusion, *L. christinae* can significantly reduce the cholesterol content in bile and inhibit the tendency of gallstone formation. From the aspect of the intestinal flora, cholelithiasis model mice had a clear intestinal flora imbalance. After administration of LAE, at the phylum level, the abundance of *Bacteroidetes* and *Firmicutes* increased and that of *Verrucomicrobia* decreased. Moreover, at the genus level, unclassified_*Porphyromonadaceae*, *Lactobacillus* and *Alloprevotella* abundances increased with a decrease in *Akkermansia*. These findings suggest that an intestinal flora imbalance may lead to gallstone formation. Thus, reducing cholesterol content in the bile by affecting the intestinal flora may be one of the mechanisms by which *L. christinae* prevents cholelithiasis.

Since the treatment and avoidance of recurring problems from CGS diseases remains a challenging and costly problem worldwide [34], a standardized extract of a natural herb with promising efficacy might provide an alternative remedy for patients during gallstone treatment or maintenance therapy. This study has important reference value for the prevention and treatment of cholelithiasis.

## Figures and Tables

**Table 1:** Weight of gallstones.

	Control check	Model	Low dose of LAE	Middle dose of LAE	High dose of LAE
Weight (g)	0	0.044 ± 0.009^**^	0.042 ± 0.006^**^	0.027 ± 0.006^**##^	0.021 ± 0.004^**##^

Data are means ± SD. *, compared to the control check; #, compared to the model group. *, #, *p* < 0.05; **, ##, *p* < 0.01.

**Table 2:** Body weight gain and main visceral indexes.

	Body weight growth (g)	Liver (%)	Gallbladder (%)
Control check	1.350 ± 0.274	4.087 ± 0.433	0.148 ± 0.033
Model	3.367 ± 0.489^**^	7.584 ± 0.627^**^	0.387 ± 0.046^**^
Low dose of LAE	3.051 ± 0.442^**^	7.673 ± 0.288^**^	0.352 ± 0.088^**^
Middle dose of LAE	2.483 ± 0.360^**##^	6.664 ± 0.396^**#^	0.296 ± 0.077^**^
High dose of LAE	2.217 ± 0.366^**##^	5.470 ± 0.647^**##^	0.226 ± 0.073^##^

Data are means ± SD. *, compared to the control check; #, compared to the model group. *, #, *p* < 0.05; **, ##, *p* < 0.01.

**Table 3:** The effect of LAE on lipid levels of plasma.

The effect of LAE on lipid levels of plasma.
	TC (mmol/L)	TBA (μmol/L)	TG (mmol/L)	LDL-C (mmol/L)	HDL-C (mmol/L)
Control check	1.10 ± 0.21	4.31 ± 0.73	0.30 ± 0.08	0.62 ± 0.04	2.62 ± 0.36
Model	7.22 ± 0.64^**^	68.70 ± 13.68^**^	1.02 ± 0.27^**^	1.06 ± 0.17^**^	1.69 ± 0.23^**^
Low dose of LAE	6.03 ± 1.42^**^	126.39 ± 18.10^**##^	0.87 ± 0.25^**^	0.99 ± 0.12^**^	2.09 ± 0.24
Middle dose of LAE	5.39 ± 0.72^**##^	96.17 ± 14.02^**^	0.79 ± 0.21^**^	0.91 ± 0.10^**^	2.25 ± 0.33^#^
High dose of LAE	4.74 ± 0.40^**##^	104.28 ± 14.59^**#^	0.61 ± 0.16^#^	0.79 ± 0.08^##^	2.25 ± 0.38^#^

Data are means ± SD. *, compared to the control check; #, compared to the model group. *, #, *p* < 0.05; **, ##, *p* < 0.01.

**Table 4:** The effect of LAE on lipid levels of bile.

	TC (mmol/l)	TBA (μmol/l)
Control check	2.30 ± 0.38	187.68 ± 10.26
Model	9.72 ± 1.71^**^	209.88 ± 7.38^*^
Low dose of LAE	8.45 ± 1.65^**^	217.55 ± 21.77
Middle dose of LAE	7.18 ± 1.34^**^	239.94 ± 25.57^*^
High dose of LAE	5.48 ± 1.55^*#^	248.40 ± 20.62^**#^

Data are means ± SD. *, compared to the control check; #, compared to the model group. *, #, *p* < 0.05; **, ##, *p* < 0.01.

**Table 5:** The effect of LAE on biochemical markers of liver injury.

	ALT (IU/L)	AST (IU/L)	AKP (IU/L)
Control check	7.12 ± 2.55	11.52 ± 6.05	57.37 ± 4.71
Model	56.02 ± 14.50^**^	19.28 ± 8.43	115.72 ± 31.26^*^
Low dose of LAE	41.01 ± 9.77^**^	17.06 ± 7.21	114.67 ± 26.81^*^
Middle dose of LAE	42.11 ± 9.73^**^	15.96 ± 6.41	107.01 ± 22.33^*^
High dose of LAE	37.03 ± 7.01^**^	12.97 ± 2.49	97.80 ± 5.23^**^

Data are means ± SD. *, compared to the control check; #, compared to the model group. *, #, *p* < 0.05; **, ##, *p* < 0.01.

**Table 6:** Detailed table of the relative abundance of the bacterial community at the phylum level.

	CK	M	JL	JM	JH
*Bacteroidetes*	70.02581	34.95608	33.77186	43.38127	39.75309
*Verrucomicrobia*	3.24671	52.50218	50.99156	32.31237	33.72824
*Firmicutes*	21.90764	8.168887	9.878015	18.59366	18.29059
*Proteobacteria*	3.78568	3.827517	4.90288	5.309093	7.446135
Other	1.034167	0.545331	0.455681	0.403603	0.78194

The relative abundance is expressed as a percentage.

**Table 7:** Detailed table of the relative abundance of the bacterial community at the genus level.

	CK	M	JL	JM	JH
*Akkermansia*	3.24671	52.50218	50.99156	32.31237	33.72824
*Lactobacillus*	9.648287	1.297114	2.045309	4.401993	1.6255
*Bacteroides*	1.310231	2.006131	6.41958	2.736214	5.280691
*Helicobacter*	2.764134	1.627525	1.196135	2.26207	4.424618
*Prevotella*	3.036439	1.044421	1.124103	2.248103	2.521237
*Alloprevotella*	6.648101	0.988195	0.287234	0.842477	1.028893
*Desulfovibrio*	0.627858	1.324684	1.640408	2.505888	2.376748
*Clostridium_XlVa*	0.826045	1.12431	0.310051	0.728852	1.021102
*Parabacteroides*	0.542998	0.571816	1.127011	0.54516	0.923596
*unclassified*_*Porphyromonadaceae*	54.14674	28.8489	22.20646	34.60356	27.82568
*unclassified_Lachnospiraceae*	5.551632	3.466279	4.227523	8.668111	9.346448
*unclassified_Bacteroidales*	2.704517	1.357682	2.441261	1.752654	1.800209
*unclassified_Ruminococcaceae*	1.691028	1.006864	1.090548	2.476535	3.122329
*unclassified_Clostridiales*	2.665578	0.173672	0.59035	0.618069	0.417885
Other	4.589703	2.660223	4.302463	3.297944	4.55683

The relative abundance is expressed as a percentage.

**Table 8:** The Adonis analysis of different groups.

Comparison	F.Model	R2	*p* Value
CK_vs_M	91.71933	0.867574	0.002
CK_vs_JL	27.04486	0.65891	0.002
CK_vs_JM	22.14715	0.612694	0.002
CK_vs_JH	26.23	0.652001	0.001
M_vs_JL	1.314809	0.085852	0.195
M_vs_JM	7.920482	0.361328	0.002
M_vs_JH	6.395739	0.313582	0.003
JL_vs_JM	3.800483	0.213504	0.008
JL_vs_JH	3.03778	0.178297	0.016
JM_vs_JH	1.435086	0.092976	0.206
